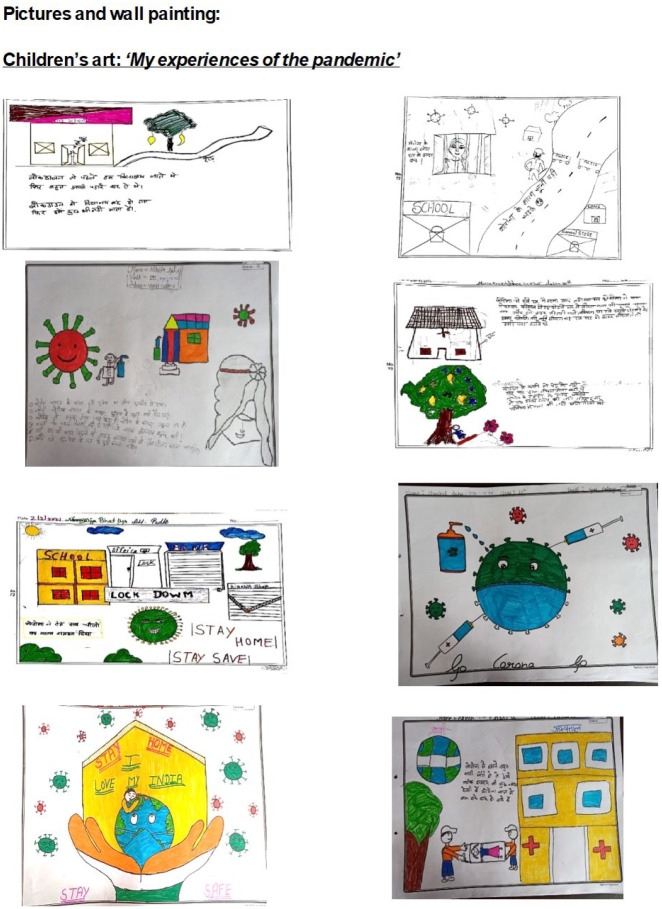# Seen and not heard: how we used visual creative activities for public health knowledge exchange with communities in rural India

**DOI:** 10.1177/17579139241252717

**Published:** 2024-11-06

**Authors:** H Chaturvedi, L Nixon, M Lakhanpaul

**Affiliations:** Population, Policy and Practice, UCL Great Ormond Street Institute of Child Health, University College London, UK; Population, Policy and Practice, UCL Great Ormond Street Institute of Child Health, University College London, UK; Population, Policy and Practice, UCL Great Ormond Street Institute of Child Health, University College London, UK

Public health is about the public, and it is recognised that they need to be part of the research process to optimise the suitability of interventions.^1^ However, communities are often not engaged with the research designed to support them, especially in low- and low-middle-income countries (LIC/LMIC).^2^ Marginalised groups are often labelled as ‘hard to reach’, but it is argued that this places blame on communities for being ‘inconvenient’, when they are often not approached with their needs in mind.^3^ Participatory research practices are a way of challenging the traditional top-down health research culture by including seldom-heard voices in research and encouraging a reciprocal relationship between researchers and communities.^4,5^ Creative activities are considered an effective, accessible and low-pressure approach to involve and engage the public in research and to minimise the researcher-community power imbalances that often come with working with marginalised groups.^6,7^ Here, we share examples of how we have used art to both learn from and share findings with rural communities in Rajasthan, India.

## Hearing from Children

India was greatly impacted by the pandemic. As children are underrepresented in research, especially those in rural areas or LIC/LMIC, we felt it was important to explore how this vulnerable population had been affected. We used a drawing activity to engage with children’s experiences of living in rural Rajasthan during the pandemic to identify and share priority research areas to ensure these children’s voices were heard and that they were not ‘left behind’.^8^ To give the children a sense of control and freedom, the field worker (H.C.) used a simple prompt – ‘draw a picture about your experiences of the pandemic’. They were encouraged to express themselves however they were most comfortable, and some chose to include writing as part of their pictures to provide additional clarity. Their drawings provided an insight into their daily lives and concerns during the pandemic; examples of their work can be found at the end of the article. Several children shared through drawings that that were not able to afford food, especially healthy food, and had concerns over their parents’ job security. They reported feeling isolated, with one child writing that she ‘felt lonely during lockdown’ and ‘used to spend a whole day just sitting under the tree’. One girl depicted her home like a jail cell, with police blocking their way out of the village. Several children also expressed concerns about missing school, as they missed their friends, were worried that they would be behind on their education and were doing more household chores instead. However, some children chose to share the more positive aspects of their experience, for example, that they were happy that they got to spend more time with their family. We also noted a clear understanding of COVID-19 public health messages. Children knew what the virus looked like, drew hospitals and vaccinations, and many chose to depict the protective measures that should be taken. This tells us that children in rural settings in India were struggling with many of the same educational, dietary and mental health concerns that children were experiencing worldwide, highlighting concerns that need to be addressed by local community programmes to ensure the children were not forgotten post-pandemic. The activity also illustrated that public health messages can reach rural communities if they are tailored and targeted effectively. Despite the challenging subject matter, the children enjoyed the activity and appreciated the opportunity to share their perspectives, illustrating how creative research activities can make serious topics accessible and how much children recognise the value of being listened to. Examples of these drawings can be found at the end of the article.

## Sharing Perspectives with Actors of Change

Listening to communities but not sharing their stories with people who can action change could be considered unethical. With their permission, the children’s drawings were shared as part of an online event focussing on child health and wellbeing in India. The event was led by an interdisciplinary panel who aimed to advocate for prioritising children in public health discussions. The event reached out to a global audience of academics, health practitioners and policymakers and involved discussions around future research and priority setting for public health recovery programmes. The artwork was used as a way of ‘bringing to life’ the discussions and to facilitate the audience to engage with the struggles the children were facing on a human level.

## Art in Community Health Promotion

For centuries, art has been an important way of narrating messages through visual stories. In LIC/LMIC, art is frequently used to communicate public health messages, especially in areas with low literacy rates or multiple dialects. We used wall art to share important public health messages on Infant and Young Child Feeding (IYCF) practices to the tribal communities we were working with. The PANChSHEEEL project was an interdisciplinary UK Research and Innovation (UKRI)-funded study bringing together partners from the UK and India to explore the Health, Education, Engineering and Environment factors that influence IYCF practices and nutrition in India.^9^ Pre-pandemic, communities were involved at various stages of the project, from informing the research design and leading the research activities as community champions, to educating the research team on how best to disseminate and implement the findings back to the community. A key aspect of this knowledge exchange was to spread awareness around the importance of hand-washing practices, especially before food preparation, to prevent transmission of infection to young children. Sharing ownership of the project with the residents, we worked with school committees, community leaders and Anganwadi (local childcare centre) workers to co-develop and implement accessible wall art to promote these health practices. The final images were painted on school and panchayat (local council) buildings in four villages in Banswara. The feedback from local groups was positive, with community leaders saying the art had sparked discussion around hygiene and that they were discussing using a similar technique for other public health messages.

## Why does this Matter?

These activities illustrate how researchers can build mutually beneficial relationships with communities using a partnership approach and utilising feasible, accessible and engaging methods. Basic creative activities can be low cost and used at all stages of the research process from setting the agenda and exploring perceptions to disseminating public health messages. It can enable a meaningful shift from communities being considered ‘hard to reach’ to them being ‘seen and heard’. However, facilitating engagement activities does not come without challenges. Building partnerships between local universities and community leaders to enable such activities to take place is needed. Often a respected, known field researcher is required build trust and facilitate the establishment of these relationships which requires time, money, commitment and practical experience.^10^ As such, it is understandable if researchers are either intimidated by projects that involve participatory methods or they embark on such projects without satisfactory expertise; this can leave everyone involved disappointed and feed into the narrative that communities are ‘hard to reach’. In our opinion, receiving iterative feedback from community partners is the best way to learn what works and how to refine one’s approach in the future. As underserved communities are often the most vulnerable to public health risks, they are the ones who researchers, policymakers and health service providers should be engaging with the most; art is a universal and accessible way to make this happen.

## Figures and Tables

**Figure:**